# Perovskite Neuromorphic Engine for Transformer Architectures

**DOI:** 10.1002/advs.202504706

**Published:** 2025-07-13

**Authors:** Zhenye Zhan, Yulu Gao, Yue Liao, Weiguang Xie, Si Liu, Xiaomu Wang

**Affiliations:** ^1^ Siyuan Laboratory Guangdong Provincial Engineering Technology Research Center of Vacuum Coating Technologies and New Energy Materials Department of Physics Jinan University Guangzhou Guangdong 510632 China; ^2^ Institute of Artificial Intelligence Beihang University Beijing 100191 China; ^3^ Hangzhou International Innovation Institute Beihang University Hangzhou 311115 China; ^4^ Department of Electronic Engineering The Chinese University of Hong Kong Hong Kong 999077 China; ^5^ School of Electronic Science and Engineering Nanjing University Nanjing 210093 China

**Keywords:** artificial neural networks, memristors, neuromorphic computing, perovskite devices

## Abstract

Memristive computing refers to the hardware implementation of artificial neural networks (ANNs) by employing memristive devices. It supports analog multiply‐and‐accumulation (MAC) operation in a compact and highly parallel manner, which can significantly enhance computing efficiency. However, applying memristive computing in advanced network structures, such as deep neural networks and multimodal networks, is inefficient because the partial analog computing requires frequently exchanging data between analog and digital domains. Here, a perovskite memristive computing unit with flexible reconfigurability and desired nonlinearity through fully vapor deposition is reported. It enables performing all the mathematical operations necessary for Transformer ANNs completely in the analog domain. A prototypical attention module is implemented by combining cells configured in different operators of dynamic MAC, activation, and softmax functions. By cascading the modules in a multi‐layer Transformer network, a neuromorphic engine is fabricated and tested RGB‐T tracking and visual question answering tasks, fully considering device non‐idealities. It is found that the network performance is close to that of operating on a graphics processing unit (GPU)‐accelerated workstation, but it consumes only 1.7% energy and increases power efficiency by 58 times. The results pave a new way toward efficient and accurate hardware memristive computing for advanced ANNs.

## Graphical abstract

This paper introduces a perovskite‐based memristive computing unit that performs all necessary operations for Transformer artificial neural networks entirely in the analog domain. The system achieves comparable performance to GPU‐based systems, but with only 1.7% of the energy consumption, boosting power efficiency by 58 times. This approach advances hardware implementation for efficient and accurate neural networks.

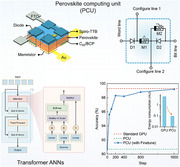

## 1 Introduction

The rapid development of modern ANNs has led to attractive progress in data‐centric applications. Unfortunately, the low communication bandwidth between data storage and processing units, known as von Neumann bottleneck in current computers, is energy inefficient and thereby not ideal for the state‐of‐the‐art ANNs.^[^ ^1^, ^2^ ^]^ Recently, memristive devices have emerged as a promising candidate for non‐von Neumann architecture.^[^ ^3^, ^4^, ^5^ ^]^ It allows storing large amounts of weights in a compact crossbar structure and directly operating the MAC through physical laws with high parallelization.^[^ ^6^, ^7^, ^8^, ^9^, ^10^, ^11^ ^]^ As a result, memristive cores were substantially employed in ANN hardware as analog accelerators to realize the most demanding and critical vector‐matrix multiplication (VMM) in a more efficient way.^[^ ^12^, ^13^, ^14^, ^15^, ^16^ ^]^

Notably, there are several other operations in addition to MAC or VMM in ANNs.^[^ ^17^, ^18^ ^]^ These operations are commonly computed by conventional processors in the digital domain. Running the memristive cores requires exchanging data between analog and digital domains. Especially, the data conversion is extremely frequent in advanced ANNs with huge numbers of layers, such as deep neural networks or Transformer neural networks. In this scenario, the overheads of high‐performance digital‐to‐analog and analog‐to‐digital converters are unaffordable in terms of power consumption and operating latency. It always results in an undesirable compromise between computing accuracy and energy efficiency.

A direct solution is to perform all the operations in the analog domain. Two prerequisites should be achieved for this purpose. First, non‐linearity is key to realizing essential functions such as activation and softmax. Second, reconfigurability is also a key requirement.^[^ ^19^ ^]^ Compared with software‐defined ANNs, hardware designs are generally difficult to modify or update without adding new physical components.^[^ ^20^ ^]^ It is thus hard to adapt various types of neural networks and tasks.^[^ ^21^, ^22^, ^23^ ^]^ Accordingly, customizable circuits can be programmed to perform specific functions using the same basic device structure, which is highly desirable for various applications.^[^ ^24^, ^25^, ^26^ ^]^ Taking all these factors into consideration, we developed a memristive perovskite computing unit (PCU) with reconfigurability and non‐linearity for hardware implementation of full analog ANNs.

## 2 Result

### 2.1 Programmable Perovskite Computing Unit

**Figure 1:**
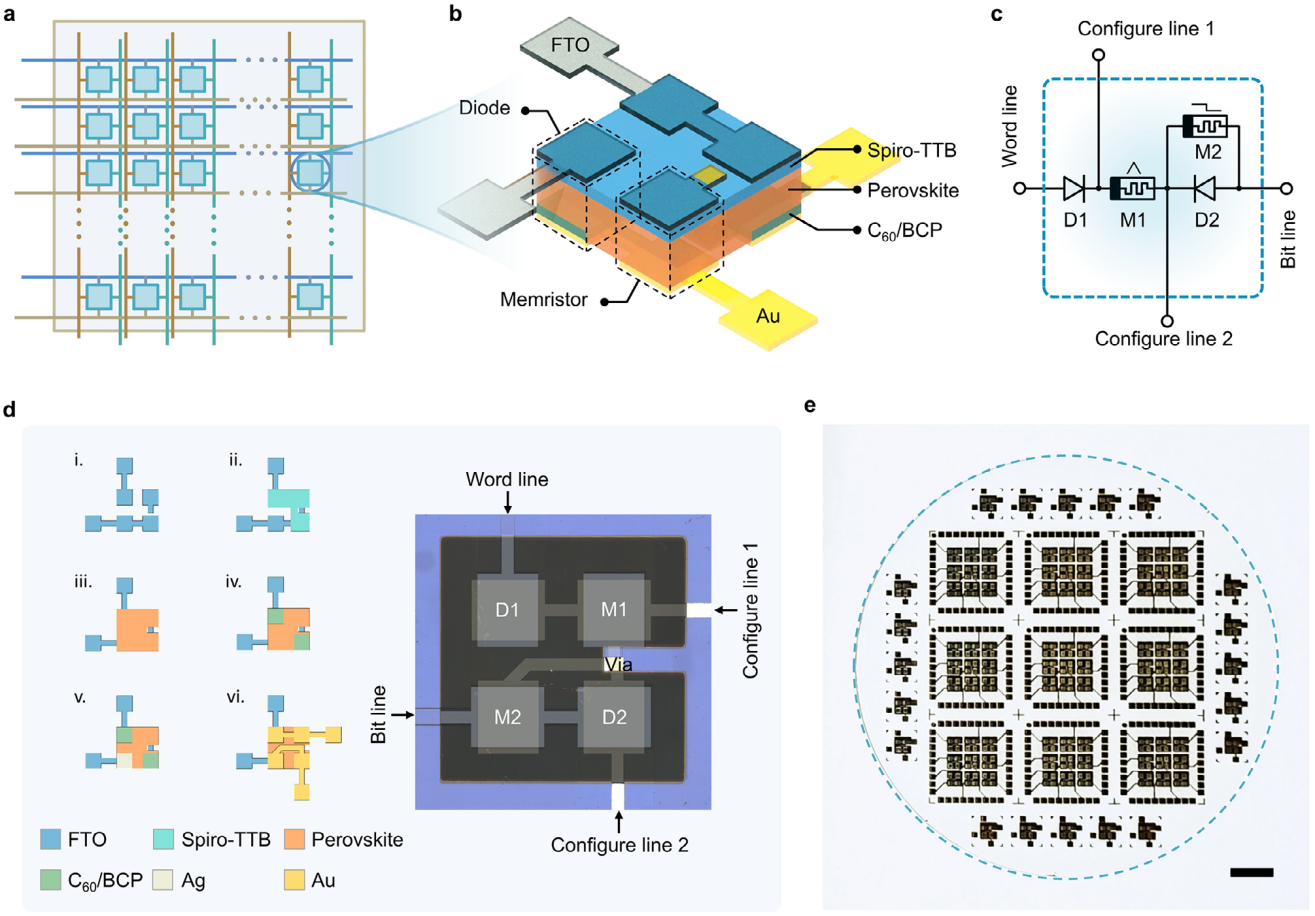
The perovskite computing unit. a) Schematic of the PCU network. b) Schematic of the programmable PCU architecture. c) Schematic symbol diagram of the PCU. D1 and D2 are diodes, used to generate nonlinear behavior for nonlinear operations. M1 is a multistate memristor used to implement VMM operations, while M2 is a binary memristor used to reconfigure the function of the circuit. The configure line 1 is used to adjust the conductance of M1, while configure line 2 switches the state of M2 to enable different operational modes. d) Left: The preparation processes of PCU and the corresponding mask area for each film layer. Right: Schematic images of a PCU. e) Optical image of a fabricated four‐inch wafer containing 101 PCUs. Scale bar: 1 cm.

**Figure** **1**a schematically illustrates the designed perovskite computing unit (PCU). The kernel of the PCU is a vapor‐deposited, superior high‐quality MAPbI_3_ perovskite active layer (see Note S1, Supporting Information for details).^[^ ^27^ ^]^ Benefiting from their promising optoelectronic properties and large‐scale integrability, perovskite memristors have been substantially studied for neuromorphic computing.^[^ ^28^, ^29^, ^30^, ^31^ ^]^ However, the traditional solution method is hard to monolithically integrate with other electronic circuits. We developed a CMOS‐compatible full vapor‐phase process that allows integration of various functional layers in the same wafer, as shown in Figure 1b. By this method, we developed prototypical PCUs that incorporated different types of components in addition to memristor arrays. Figure 1c schematically summarizes the structure of this PCU. It is comprised of 4 adjacent components, including two hetero‐stacked diodes and two memristors. First, two back‐to‐back diodes (D1, D2 in Figure 1c) provide strong non‐linearity. They were fabricated by stacking of FTO top electrode/Spiro‐TTB hole transport layer/perovskite/C_60_/BCP electron transport layer/Au bottom electrode. Second, one multi‐state memristor (M1 in Figure 1c) is used to realize the basic memristive function. It is designed as a stacked structure of FTO top electrode/Spiro‐TTB/perovskite/Au bottom electrode. Third, another binary memristor (M2 in Figure 1c) works as a switch. It is composed of bare perovskite thin film sandwiched between FTO and Ag electrodes, which is in parallel with a backward diode. The word line and bit line are integral to the crossbar structure, enabling the device to perform complex computations by interconnecting the PCUs in various combinations. The configure line 1 and line 2 are used to apply necessary voltage biases to set the resistance states of memristors M1 and M2. This arrangement allows electrical configuration of the PCU to perform specific functions without any hardware changes.

Notably, all the functional layers are prepared by a similar vapor‐phase deposition, ensuring precise control of layer thickness and device uniformity in wafer‐level manufacturing. The standard deviations of the absorption edge and X‐ray diffraction (XRD) characterization at different locations on the 4‐inch perovskite film are only 0.63 nm and 0.01°, respectively (Figure S2, Supporting Information), and this essentially meets the demands of high‐performance computing systems. As a demonstration of the scalability, 101 PCUs were prepared on a 4‐inch glass substrate containing nine 3 × 3 PCU arrays and another 20 individual PCUs (Figure 1e).

**Figure 2:**
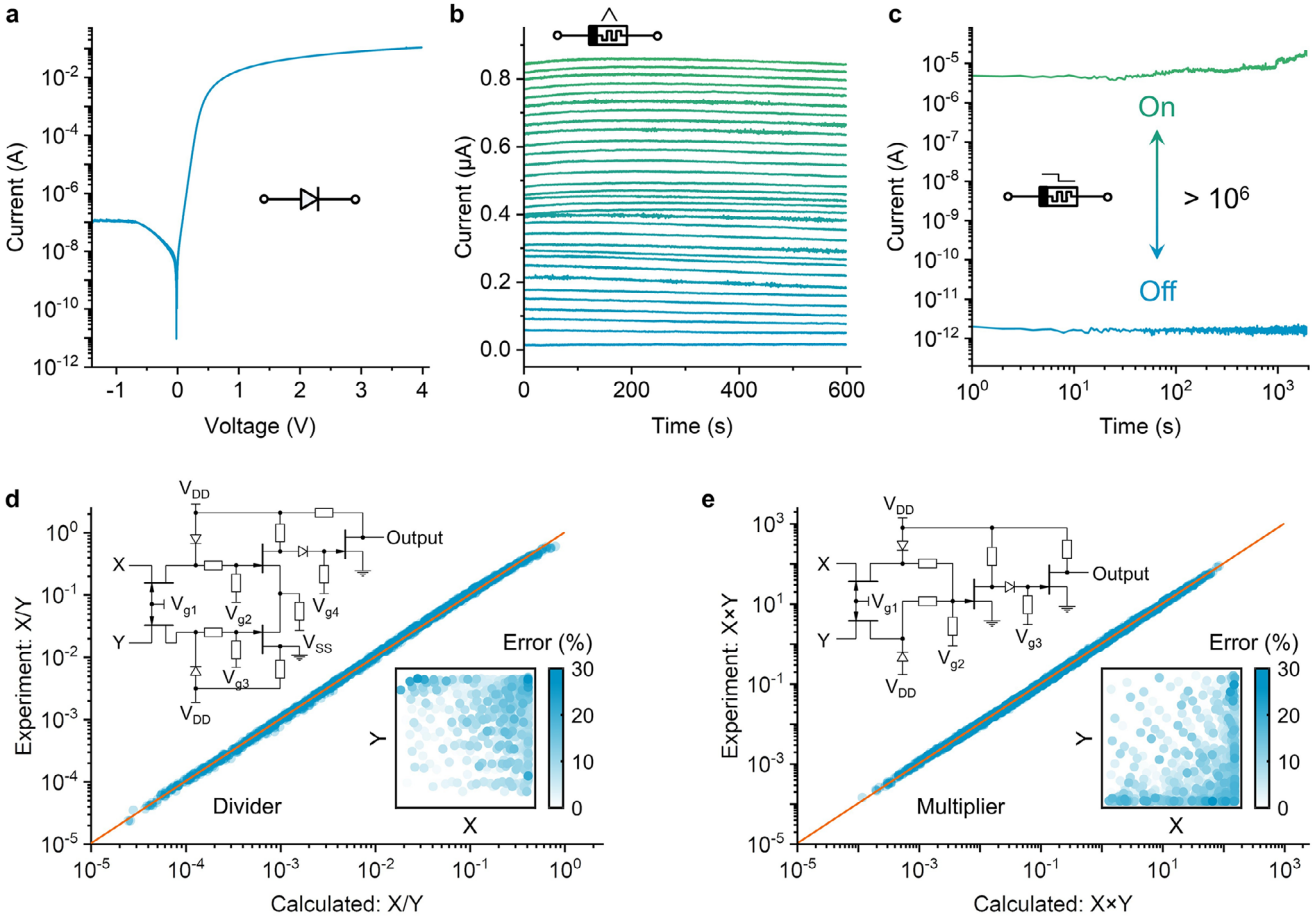
Electronic properties of PCU. a) The current‐voltage characteristic of the perovskite diode in PCU. b) Retention performance of the of the multi‐state memristor in PCU. The conductance can be continuously modulated. Measured at +0.01 V. c) Retention performance of the LRS (On) and HRS (Off) of a binary memristor read at +0.1 V. d,e) Comparison of experimental results and computational true‐value for 10 dividers d) and 10 multipliers e) (Insets: top left: circuit diagram, bottom right: error as function of two operator inputs).

After fabricating the PCU, we first characterize the electrical performance of each individual component. The prepared perovskite diode (D1 & D2 in PCU) shows a typical rectification behavior as shown in **Figure** **2**a. The *I–V* characteristics can be described by $I=I_{0}\left[\exp(\frac{q(V-IR_{s})}{nK_{B}T})-1\right]$. *I*_0_ is the reverse saturation current, *q* is the elementary charge, *R_s_* is the series resistance. In the forward bias case, there is an approximately exponential relationship between the channel current and applied bias voltage. This non‐linearity is elemental in achieving arbitrary arithmetic operations such as multiplication and division.

M1 in PCU is a multistate memristor. Figure 2b shows its continuous conductance modulation performance. This memristor behavior results from a charge‐trapping/detrapping mechanism.^[^ ^32^ ^]^ Briefly, charge can either be injected into or withdrawn from the trapped states under an external electric field, which adjusts the barrier at the MAPbI_3_/Au interface and changes the conductivity. Figure S4 (Supporting Information) characterizes the memristive characteristics of the multi‐state memristor. The multi‐state memristor exhibits exceptional state retention, with each distinct resistive state maintained stably for over 600 s, demonstrating its potential for reliable multi‐level data storage applications. Long‐term stable cycling operation based on advanced research of halide perovskites also confirms this.^[^ ^28^ ^]^ This robust fine‐tuned control over conductance levels allows us to implement VMM for neural networks.

M2 in PCU is a binary memristor. It is the key component for reconfigurability. It can control the current path by switching the conductance state. With a low resistance state (LRS), it shortcuts the diode. By contrast, with a high resistance state (HRS), it has no influence on the connection of the backward diode in the circuit. Figure S5 (Supporting Information) characterizes the resistive switching of the binary memristor by hysteresis I–V sweeping. The device demonstrates a reliable and stable performance with LRS/HRS current ratio of over 10^6^ and retention time exceeding 10^3^ s, under a continuous reading bias of +0.1 V (Figure 2c). We attribute the reliable resistance switching observed in the FTO/MAPbI_3_ perovskite/Ag binary memristor to the presence of conductive filaments in the MAPbI_3_ perovskites.^[^ ^33^, ^34^, ^35^ ^]^ The specific working mechanisms of binary and multi‐state memristors are discussed in Note S2 (Supporting Information).

We then exemplified multiplication and division by PCU, as they are the backbone of all other required operations. We programmed both M1 and M2 to the LRS state in the PCU to utilize the nonlinear relationship of perovskite diodes. This enabled hardware implementations of dividers and multipliers in the analog domain. The key to this implementation relies on the exponential *I*–*V* curve, which transforms division and multiplication operations to subtraction and addition operations in the logarithmic domain. Figure 2d,e illustrates the specific implementation and measured results for the divider and multiplier, respectively. Each multiplier and divider requires three PCUs (See Experimental Section for details). A schematic illustrating the interconnection of PCUs to form the divider and multiplier is included in Figure S7 (Supporting Information). The average relative error of ten dividers (multipliers) based on 10 devices is 5.66% (6.61%).

### 2.2 Attention Module Implemented by PCU

**Figure 3:**
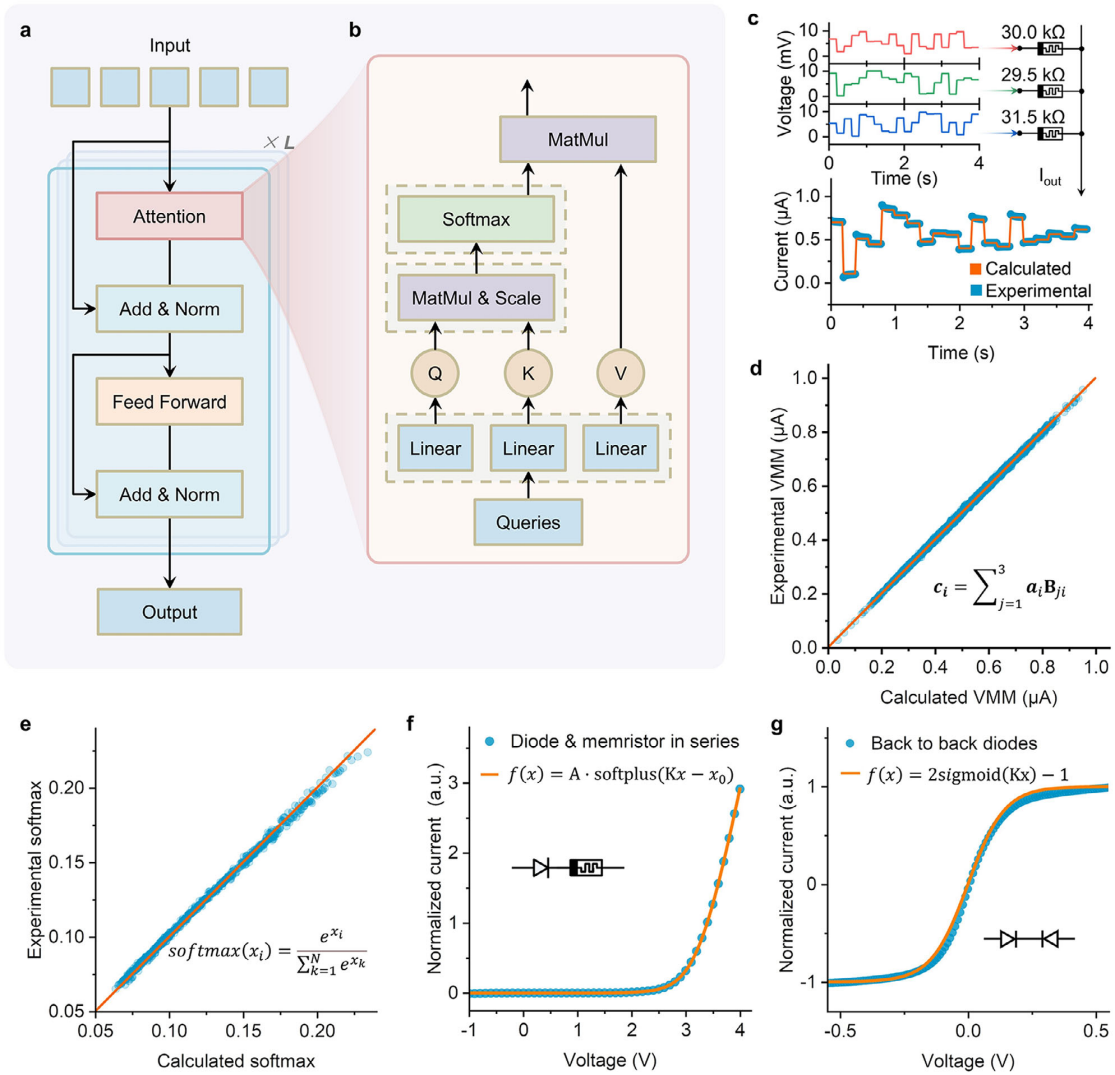
Attention module implemented by PCU. a,b) The computing flow chart of the basic blocks of the Transformer network a) and Scaled Dot‐Product Attention module b). c) Illustration of the VMM operation involving a 1 × 3 vector and a 3 × 20 matrix implemented using a 1 × 3 multi‐state memristor crossbar array. d) Comparison of VMM measurement results between expected and measured values derived from a 1 × 3 multi‐state memristor crossbar array. e) Comparison of experimental results and computational results for the softmax function. Insets: circuit diagram. f) The softplus activation function implementation based on the perovskite diode and programmed memristor. g) The sigmoid activation function implementation based on the two back‐to‐back connected perovskite diodes.

With these basic devices, almost all the analog operators necessary for advanced ANNs can be performed by properly configuring and cascading PCUs in certain structures. As aforementioned, this flexibility is highly suitable for modern ANNs. We thereby demonstrated an instance of the implementation of Transformer network. Transformer networks have revolutionized deep learning by introducing attention mechanisms that allow models to capture complex relationships within data, significantly advancing fields like natural language processing and computer vision.^[^ ^36^, ^37^, ^38^ ^]^ The kernel building‐block of a Transformer network (**Figure** **3**a) is the so‐called attention module (Figure 3b). Compared with conventional artificial synapses, it contains more complex arithmetic operations in addition to the MAC operator.

We demonstrated the realization of the entire attention module based on the PCUs. As shown in Figure 3b, the first stage of the attention module is a linear VMM of the input vector queries and the weight matrix. We achieved these structures by configuring PCUs into a memristor crossbar. For this purpose, in a PCU crossbar, we set all the M2 to LRS and store weights in M1. Notably, diode D1 acts as a natural selector for the memristor. Figure 3c shows the results of an example for a 1 × 3 vector (mimics the weight of an ANN) multiplying a 3 × 20 matrix (mimics the input). There is a good agreement between the experimental result and the mathematical true value. To provide further quantitative analysis, the operations between a 1×3 vector and a 3 × 1000 matrix were carried out, and the result distribution was plotted in Figure 3d. By comparing the experimental data with the expected mathematical calculations, the average relative error is only 0.78%, showing the effectiveness and reliability of this system in practical applications. The second step of the attention module is the softmax calculation. In this process, the calculated product matrices (Q, K, V) are scaled (by dividing them by the square root of the key dimension) and fed into a softmax function defined as $softmax\left(x_{i}\right)=\frac{e^{x_{i}}}{\sum_{k=1}^{N}e^{x_{k}}}\;$. The whole function reads $attention\;\left(Q,K,V\right)=softmax\left(\frac{QK^{T}}{\sqrt{d}}\right)V$, where $1/\sqrt{d}$ serves as a constant scaling factor. For this softmax hardware, multipliers, diode exponential operators, and dividers are configured for the softmax function. The average relative error of softmax calculations based on 10 hardware units is 1.31% (Figure 3e).

### 2.3 Programmable Activation Function

Activation is another essential step in neural networks. It introduces non‐linearity to the model, enabling the network to learn complex patterns and relationships from the data that linear transformations cannot capture.^[^ ^39^, ^40^, ^41^ ^]^ There are various types of activation functions designed for different models. Here, we demonstrated the construction of two popular activation functions by PCUs.

We first demonstrated the implementation of the softplus activation function, which is a variant of the most popular ReLU function. A single diode within the PCU (Figure 3f) simply forms the softplus function. This configuration is accomplished by programming the M2 to LRS and setting M1 ≈10 kΩ. The *I–V* characteristics of the diode under a large voltage mimic the function of *f*(*x*)  =  *A* · *softplus*(*Kx*), where the softplus function is defined as *softplus*(*x*)  =  *ln*(1 + *e^x^*). This softplus function maps all inputs to a range between zero and positive infinity. It provides a smooth, continuous transition at the whole input range, thereby avoiding issues related to non‐differentiability. This characteristic enhances the gradient flow during backpropagation, potentially leading to improved training dynamics in neural network architectures.

Due to the reconfigurable nature of the PCUs, we can program the hardware into different activation functions. Here, we also showcased the implementation of the Gaussian error linear unit (GELU) activation function.^[^ ^42^ ^]^ It provides a smoother activation curve that better captures complex patterns and improves training dynamics. This is particularly beneficial in large‐scale networks such as natural language processing tasks under the Transformer model. The GELU function is mathematically approximated by *x* · *sigmoid*(1.702*x*). The kernel operator of GELU is a sigmoid function implemented by two back‐to‐back connected perovskite diodes (Figure 3g). We achieved it by programming the M1 to LRS and the M2 to HRS in the PCU. The *I–V* characteristics of back‐to‐back diodes fit the form of *f* (*x*) =  2*sigmoid*(*Kx*) − 1, where the sigmoid function is defined as $sigmoid\;\left(x\right)=\frac{1}{1+e^{-x}}$. The detailed configuration parameters, including target functions, configuration voltages, and corresponding memristor states, are provided in Table S1 (Supporting Information).

### 2.4 Multimodal Deep Learning with Perovskite Computing Unit

We next move to discuss employing PCUs in multimodal deep learning. Deep learning has evolved from single‐modal to multimodal paradigms, reflecting how humans integrate visual, auditory, and tactile inputs to interpret the world. Traditional visual tasks often rely on convolutional neural networks, which benefit from memristive hardware accelerators to improve efficiency. However, the growing focus on multimodal tasks, essential for applications like autonomous systems and human–computer interaction, introduces greater complexity. Models must now combine information from different sensory sources to enable more context‐aware decision‐making. Attention‐based architectures like Transformers excel in such tasks by capturing intricate relationships between modalities, outperforming convolutional networks in areas such as visual question answering (VQA). Despite their effectiveness, Transformers require more complex operations, including dynamic multiplication and normalization, which pose hardware challenges. To address this challenge, we developed a PCU‐based hardware to implement Transformers by facilitating all essential steps (including attention, add & normalize) and feed forward layers, entirely in the analog domain.

**Figure 4:**
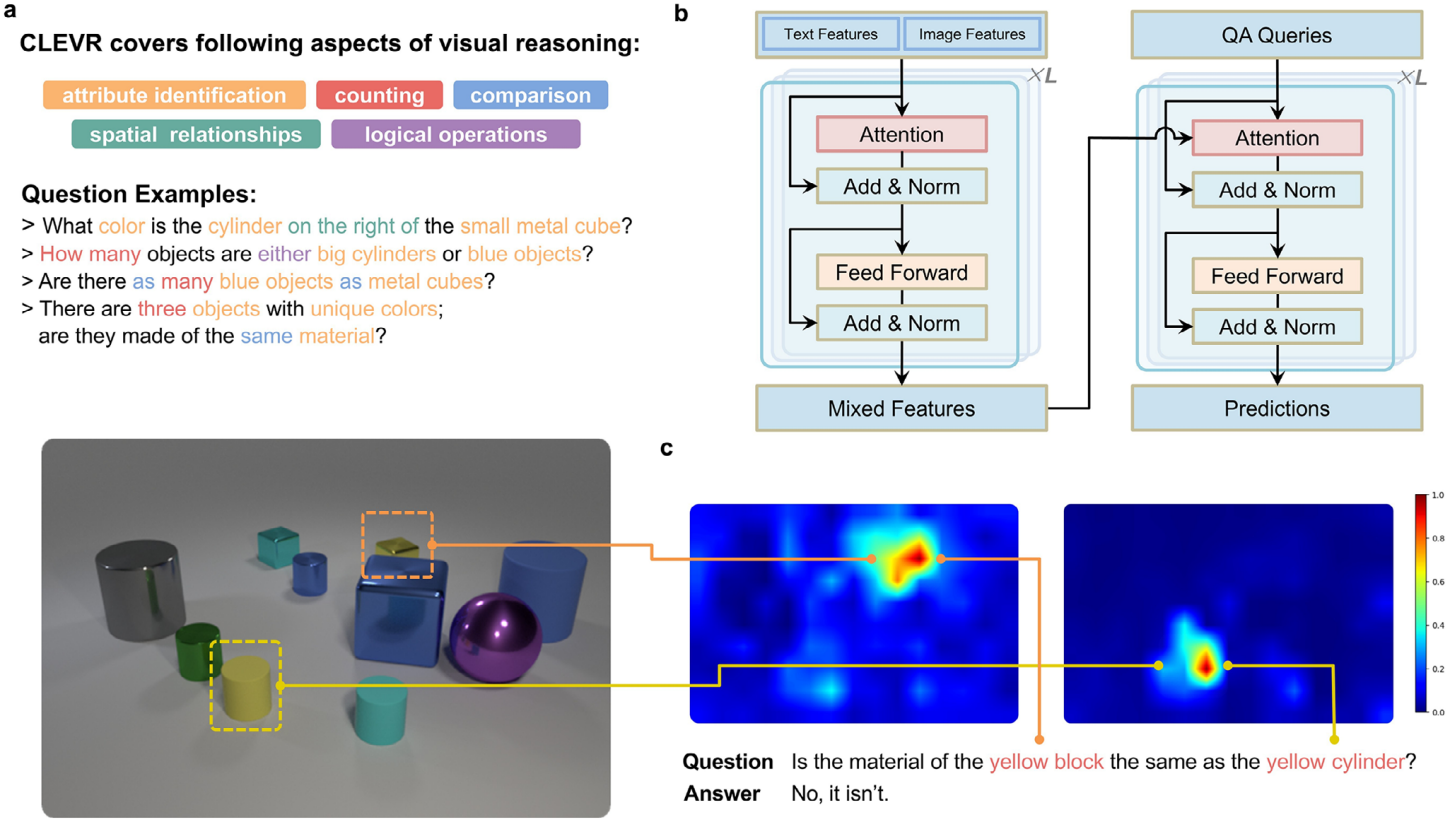
Multimodal deep learning with perovskite computing units. a) Visual question answering (VQA) task based on the CLEVR dataset. b) The Transformer architecture used, where both the encoder and decoder are constructed by memristive PCUs. c) Attention map illustrating language and visual interaction. The results show the correct answer and higher responses at image locations corresponding to the object described by the language input.

To evaluate the PCU‐based hardware for multimodal tasks, we focused on the VQA task using the widely adopted synthetic dataset CLEVR.^[^ ^43^ ^]^ As shown in **Figure** **4**a, this task requires the model to answer questions based on images, covering topics such as object attribute recognition, quantity counting, comparisons, and spatial relationships. This task effectively benchmarks the capability of Transformers in processing multimodal information. We employed an MDETR^[^ ^44^ ^]^ architecture as shown in Figure 4b. The key components are a Transformer encoder and decoder. The encoder deeply fuses visual and textual features, while the decoder generates the final answers from the fused features. We performed a system‐level simulation to test the hardware implementation of this VQA task. At first, we experimentally built all the necessary modules of the MDETR architecture using PCUs. And then, the measured figures of merit, together with device non‐idealities, were used to simulate a full Transformer network, which consists of six encoder layers as well as six decoder layers (see Experimental Section for details). Both the encoders and decoders rely on the attention mechanism. These processes involve complex computational operations, including dynamic multiplication, exponential functions, and division. All the operations can be efficiently implemented by analog computation using our hardware. We directly measured the device parameters from in‐situ experiments to ensure the accuracy of the simulation results.

The effectiveness of the Transformer network is illustrated in Figure 4. It shows a typical VQA example. The Transformer network correctly answers the textual question. It is also able to accurately capture the complex interactions between textual and visual modalities during encoding. For instance, the intermediate results shown in Figure 4c visualize the encoder‐layer attention heatmap. The map shows the weights between each text token and every image patch, highlighting the spatial regions the model attends to when answering the question. These results verified the model's ability to utilize and fuse multimodal information. Overall, the PCU hardware‐based emulation presents an accuracy of 99.2% in the VQA task. It is comparable to the workstation‐based results (99.3%), confirming the feasibility of our approach in multimodal applications.

In addition to VQA, our approach was also validated on another RGB‐T tracking task (see Experimental Section for details). This task involves tracking targets using both thermal infrared and visible images, often under challenging conditions such as varying illumination. Different from the VQA task, the RGB‐T uses the GELU as the activation function. We thus programmed the PCU into the GELU form as aforementioned. Our method, based on the TBSI structure ^[^ ^45^ ^]^ achieved a precision of 62.1% in the PCU hardware‐based emulation on the LasHeR dataset.^[^ ^46^ ^]^ It is comparable to the original TBSI network run on a workstation, which reported a precision of 64.1% (see Figure S9, Supporting Information for details). Additionally, our approach demonstrated a normalized precision of 58.7%, comparable to the original TBSI of 60.0%. These findings highlight the important role played by the reconfigurability of PCUs. It paves the way for the hardware implementation of a general neural network processor.

### 2.5 Hardware‐Aware Offline Fine‐Tuning

We finally considered the correction of non‐ideal characteristics of PCUs. Accuracy is a fundamental challenge in analog computing. Compared with digital data, analog signals have lower noise tolerance and higher errors. In particular, errors may accumulate during forward propagation, causing significant deviations from expected results.^[^ ^47^, ^48^ ^]^

**Figure 5:**
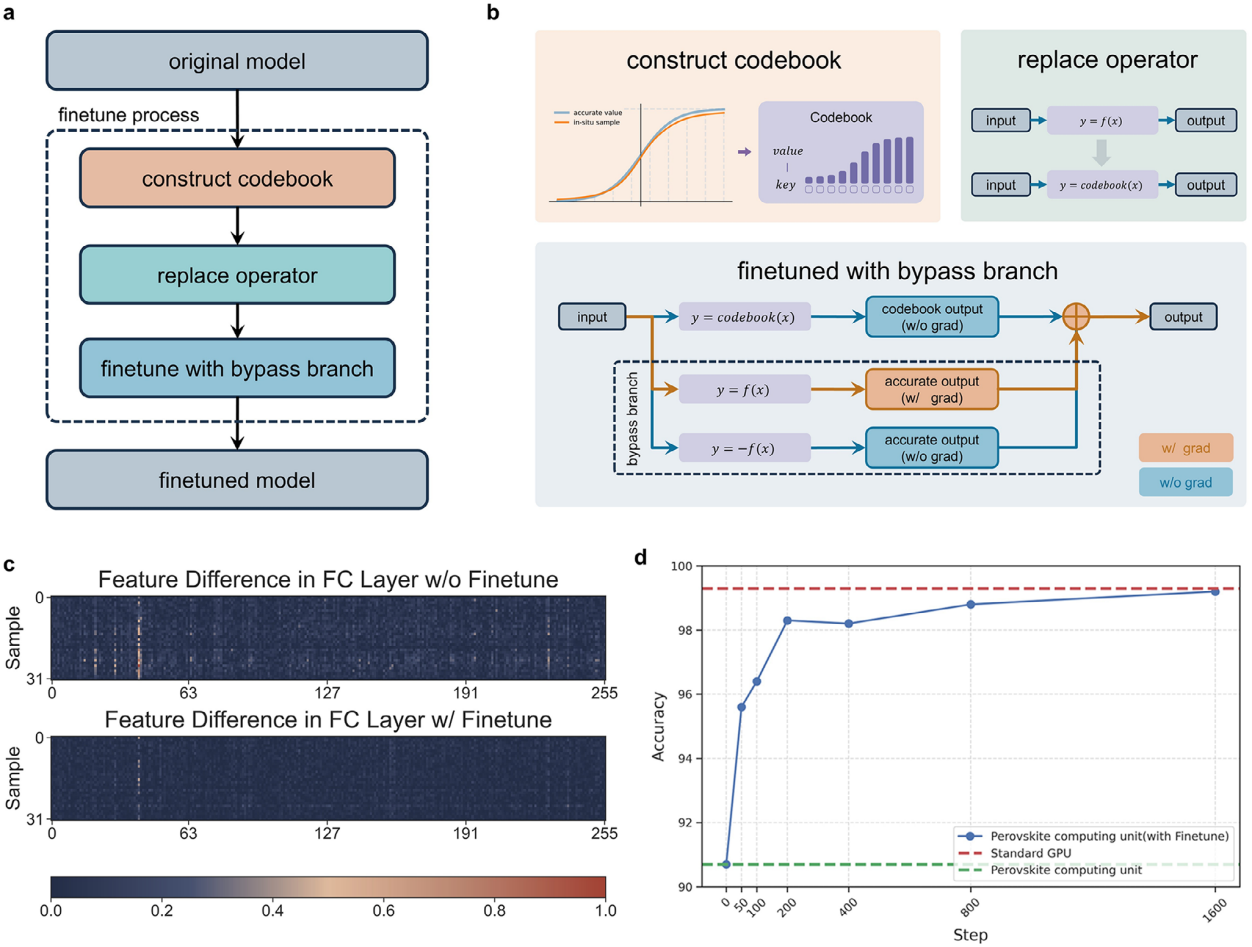
Hardware‐Aware Offline Fine‐Tuning. a) Flowchart of the Hardware‐Aware Offline Fine‐Tuning process in our experiments. b) Detailed implementation of Hardware‐Aware Offline Fine‐Tuning. This process consists of three stages: 1) Construct Codebook, where in‐situ data is sampled to build a codebook used for both training and testing; 2) Replace Operator, where the model's original operators are replaced with those from the codebook to generate outputs; 3) Fine‐tune with Bypass Branch, where simulated data (from the codebook branch) and accurate data (from the bypass branch) are used to generate outputs, and the final outputs are composed by the codebook branch, while gradient backpropagation is executed by the bypass branch. c) Comparison of feature layer outputs, showing that fine‐tuned models closely align with the original outputs, whereas non‐fine‐tuned models exhibit significant discrepancies. Color bar: normalized error between GPU‐based model and the PCU implemented model. d) Fine‐tuning curves demonstrating that our proposed Hardware‐Aware Offline Fine‐Tuning method enables the model to quickly adapt to the hardware, achieving accuracy close to that of GPU‐based models.

Reducing performance loss is aroused from the software–hardware discrepancies typically involves in situ training or online fine‐tuning during deployment. However, these methods often require substantial additional circuitry, increased system complexity and training costs. Existing online training approaches are limited by network scale and necessitate new optimization algorithms. To bridge this gap, we proposed a Hardware‐Aware Offline Fine‐Tuning strategy. This approach aims to minimize accuracy loss when migrating models from GPU training to memristive hardware. **Figure** **5**a shows the flowchart of the Hardware‐Aware Offline Fine‐Tuning process in our experiments. The key idea is inserting three additional steps in the network deployment as shown in Figure 5b.

First, we measured operational errors for the full set of PCU analog operators, namely the differences between experimental and expected outputs. According to these measurements, we generated a codebook that maps the input to the real experimental output for each analog operator. Second, during the training process, the output of each analog operator is corrected according to the input data and the codebook. In this way, the network could more accurately simulate the hardware behavior. Notably, the errors may break the well‐defined gradient of each analog component. We thus incorporated bypass branches to ensure smooth gradient propagation, enabling all model parameters to remain trainable. Specifically, we added additional surrogate paths for gradient flow as shown in Figure 5b. For arbitrary operation *y*  =  *f*(*x*), we replace it with *y*  =  *codebook*(*x*) + *f*(*x*) − sg(*f*(*x*)), where *sg*() stands for the stop‐gradient operator that returns the same values as its input but does not propagate gradients.^[^ ^49^ ^]^ This approach allows the model to simultaneously utilize hardware outputs and maintain effective gradient transmission during backpropagation.

The whole strategy effectively corrects the non‐idealities of all analog operators. We verified it by the aforementioned MDETR model on the CLEVR dataset for the VQA task. The GPU‐trained model achieved an accuracy of 99.3%, serving as our baseline. Without any corrections applied to the PCU, the model's accuracy severely dropped to 90.7%. After applying our Hardware‐Aware Offline Fine‐Tuning, the model's accuracy reached 99.2% after just 1600 iterations, closely matching the baseline performance (Figure 5d). This fine‐tuning required significantly fewer iterations than initial training, demonstrating high efficiency. This result verifies the system's ability to emulate ideal computations despite analog nonidealities that typically affect accuracy, showcasing the effectiveness of our approach in mitigating their impact. In contrast, models fine‐tuned without gradient incorporation achieved only 96.4% accuracy, highlighting the necessity of gradient flow in the fine‐tuning process. Figure 5c compares feature layer outputs. It clearly shows that fine‐tuned models closely align with the original outputs, whereas non‐fine‐tuned models exhibit obvious discrepancies.

## 3 Conclusion

We have experimentally demonstrated PCU hardware for the Transformer network. It exhibits superior performance in processing data with multimodal interaction and fusion. By correcting the device's nonidealities through software‐hardware co‐design, the prototypical neuromorphic engine made of PCU attention modules demonstrates high accuracy in executing typical tasks of the Transformer architecture. It is completely comparable to GPU‐accelerated workstations.

In addition, this neuromorphic computing engine can also enhance computational efficiency. Under the same settings, the GPU‐based Transformer block requires 5.37 mJ energy for each operation, whereas using the memristive MAC accelerator reduces the consumption to 0.32 mJ. Remarkably, our PCU‐based counterpart only consumes 0.09 mJ per operation, which is only 1.7% of the GPU used (See Note S4 and Figure S8, Supporting Information for details). Even compared with pure memristive accelerators, the PCU hardware still saves energy up to 71.9%, owing to the full set of analog operations that dispense with frequent analog‐digital data conversions.

Overall, the PCU hardware is of high accuracy, low power consumption, highly compact, and low‐cost features. This result highlights the potential of memristive computing in advanced neural network architectures, offering a promising route for more scalable and energy‐efficient multimodal computing solutions.

## 4 Experimental Section

### Materials and Characterization

All of the chemicals used in this study were obtained commercially and used as received. Methylammonium iodide (CH_3_NH_3_I, MAI) (99.8%) was purchased from TCI. 2,9‐dimethyl‐4,7‐diphenyl‐1,10‐phenanthroline (BCP) was purchased from Xi'an Polymer Light Technology Corp. 2,2′,7,7′‐tetra(N,N‐di‐tolyl)amino‐9,9‐spirobifluorene (Spiro‐TTB), Lead(II) iodide (PbI_2_) (99.99%) and C_60_ were purchased from Advanced Election Technology Co., Ltd. Scanning electron microscopy (SEM) images were acquired through an FEI Quanta 450 FEG field emission scanning electron microscope. Ultraviolet–Visible (UV–Vis) absorption spectra were measured using SHIMADZU UV‐2600. XRD spectra were acquired using a BRUKER D8‐Advance equipped with Cu Kα1 irradiation (λ = 1.5406 Å).

### Device Fabrication

PCUs were fabricated using a vapor deposition process. This process involves stacking thin films according to different component device structures to achieve the desired device properties. The binary memristors were fabricated with a device configuration of FTO/Perovskite/Ag. The multi‐state memristors were fabricated with a device configuration of FTO/Spiro‐TTB/Perovskite/Au. The perovskite diodes were fabricated with a device configuration of FTO/Spiro‐TTB/Perovskite/C_60_/BCP/Au. Taking the diode as an example, the devices were prepared on the FTO substrates, which were prepatterned, cleaned, and treated with oxygen plasma successively. A 6 nm‐thick layer of Spiro‐TTB was first deposited by thermal evaporation at a rate of 0.1 Å s^−1^. The perovskite film was prepared using a layer‐by‐layer sequential vapor deposition method.^[^ ^50^ ^]^ Specifically, a 250 nm‐thick layer of PbI₂ was initially deposited by thermal evaporation at a rate of 1 Å s^−1^. And then MAI powder was sublimed onto the PbI_2_ layer and reacted in situ to form a perovskite thin film in a vacuum oven preheated to 180 °C. After cooling to room temperature, the perovskite was cleaned by spinning isopropyl alcohol and annealed at 100 °C for 10 min. Subsequently, 23 nm of C_60_ was thermally evaporated onto the perovskite film at a rate of ≈0.1 Å s^−1^. An 8 nm‐thick BCP layer was then deposited at a rate of ≈0.08 Å s^−1^. Finally, the metal electrode was thermally evaporated through a shadow mask to complete the device. To enable monolithic integration of different device structures within a PCU, different metal masks were employed during the evaporation deposition process. Further details of fabrication steps can be found in Note S1 (Supporting Information).

### Electrical Characterization

The devices were characterized without encapsulation under atmospheric conditions and at ambient temperature. Current‐voltage characteristics of PCU elements and circuits were measured using Keithley 2612A Source Meters. The precise resistance states of multi‐state memristors were achieved via the Incremental Step Pulse with Verify Algorithm (initial amplitude ±0.2 V, step size ±0.1 V, width 0.02 s interleaved with +0.01 V read voltages).^[^ ^51^ ^]^ The VMM measurement of the 1 × 3 multi‐state memristor crossbar array was conducted using a semiconductor parameter analyzer (FS‐Pro, PRIMARIUS). The interconnection and switching between PCUs for different functions were carried out through the home‐built multi‐channel scanning system (Keithley 3706A switch/multimeter with inserted Keithley 3730 Matrix card). The device, under short‐term tests conducted in ambient conditions (≈25 °C, 30–50% RH), showed no obvious degradation, indicating stability under mild conditions. For stability under extreme conditions (e.g., high temperature/humidity) in practical applications, improvement methods have been demonstrated, such as compositional doping and passivation strategies, as demonstrated in recent studies.^[^ ^52^, ^53^, ^54^ ^]^

### Hardware Simulation

The hardware behavior was simulated based on measured device characteristics. First, discrete PCU test structures were constructed to implement and characterize analog computing operators. Then integrated experimentally measured transfer characteristics and device‐level nonidealities into our system‐level simulation framework. For the weights in the network, the approach was followed as described in ref.,^[^ ^48^ ^]^ modeling variations using a Gaussian distribution with zero mean and a standard deviation σ of 0.027367 in the experiments. The dynamic MAC, activation, and softmax operations in the network are simulated using empirically measured input‐output mappings to accurately represent hardware behavior. Again, Gaussian‐distributed noise was purposely added during the simulation to reflect the device's nonidealities. The means and deviations were obtained from statistics of experiments.

### VQA Task

1) *Dataset*: The experiments were conducted on the CLEVR dataset, which consists of 3D‐rendered environments. Each environment was paired with questions related to the visible objects, formulated using a standardized set of templates. *2) Evaluation Metric*: Model performance was evaluated based on accuracy on the CLEVR dataset. *3) Network Structure*: The model was based on the MDETR framework and includes a ResNet‐18^[^ ^55^ ^]^ backbone for image feature extraction, as well as DistilRoBERTa^[^ ^56^ ^]^ for text encoding. The final Transformer architecture mirrors that of DETR, consisting of 6 encoder layers and 6 decoder layers. The number of object queries is set to 25. *4) Training Process*: Starting with a pretrained MDETR model, the network was adapted by replacing ReLU activations with softplus. This adaptation was performed using a learning rate of 1 × 10^−5^, a batch size of 64, and over 10 000 steps. In the second stage, Hardware‐Aware Offline Fine‐Tuning was employed to adapt the model to the hardware. In situ measured data was incorporated of linear, activation, multiplication, and softmax operations into the training process. The exact multi‐task loss was employed by the original MDETR model—i.e., an L1 + GIoU loss for bounding‐box regression, cross‐entropy for class/answer prediction, and contrastive alignment for text‐vision pairs. Using the checkpoint from the first stage, this loss was optimized using AdamW (weight decay = 1 × 10^−4^) with an initial learning rate of 1 × 10^−5^, a batch size of 64, and 1600 gradient steps.

### RGB‐T Tracking Task

*1) Dataset*: Experiments were conducted on the LasHeR RGB‐T tracking benchmark. LasHeR was a large‐scale and high‐diversity dataset for RGB‐T tracking. It comprises 1224 aligned visible and thermal infrared video pairs, totaling over 730 000 frames with high‐quality bounding box annotations. It captures a wide range of object categories, viewpoints, scenes, and environmental conditions across different times and weather conditions. *2) Evaluation Metric*: Two widely adopted metrics were utilized to evaluate the method. The precision rate measures the percentage of frames where the distance between the predicted position and the ground truth was less than a certain threshold. To address the variability in target sizes, the normalized precision rate was calculated by normalizing the precision rate based on the size of the ground truth bounding box. *3) Network Structure*: The model was based on the TBSI framework, employing a ViT‐Tiny backbone.^[^ ^38^ ^]^ The fusion module, consisting of a single‐layer Transformer designed for template interaction, was integrated into the 4th, 7th, and 10th layers of the ViT backbone. RGB and thermal infrared data were processed through separate branches, and multimodal interaction was achieved via template interaction in the fusion module. The features were then merged and passed through the tracking head for prediction. *4) Training Process*: Starting with a pretrained ViT‐T trained on ImageNet,^[^ ^57^ ^]^ the network was adapted by replacing Layer Normalization with Batch Normalization.^[^ ^58^ ^]^ This adaptation was performed using a learning rate of 4 × 10^−4^, a batch size of 32, over 50 epochs. In the second stage, Hardware‐Aware Offline Fine‐Tuning was employed by continuing from the checkpoint of the first stage. The model was fine‐tuned over 10 epochs with an initial learning rate of 4 × 10^−4^, which was reduced at the 8th epoch to further optimize the model and obtain the final results. To further refine the model's activation functions, a final fine‐tuning step was performed for 1 epoch.

### Statistics Analysis

The data obtained from *I–V* measurements, SEM, XRD, and UV–Vis absorption spectra were the original data without normalization. The activation functions implemented by the PCU were normalized to their respective ranges. All statistical analyses were performed with Python.

## Conflict of Interest

The authors declare no conflict of interest.

## Author Contributions

Z.Z. and Y.G. contributed equally to this work. X.W. conceived the project. Z.Z. fabricated and measured the samples. Y.G. carried out the ANN modeling and application. Y.L. helped perform ANN modeling and contributed to the data processing. X.W., W.X., and S.L. analysed the data and wrote the manuscript. X.W., W.X., and S.L. supervised the research. All authors discussed the obtained results.

## Supporting information



Supporting Information

## Data Availability

The data that support the findings of this study are available from the corresponding author upon reasonable request.
