# Supplementary material for: Perovskite Neuromorphic Engine for Transformer Architectures
Source: Adv Sci (Weinh). 2025 Jul 13;12(33):e04706. doi: 10.1002/advs.202504706 (PMC12412470; doi:10.1002/advs.202504706)
Supplement: Supplementary file 1 — Supporting Information [file ADVS-12-e04706-s001.pdf]

ADVANCED SCIENCE
Open Access

Supporting Information

for *Adv. Sci.*, DOI 10.1002/advs.202504706

Perovskite Neuromorphic Engine for Transformer Architectures

Zhenye Zhan, Yulu Gao, Yue Liao, Weiguang Xie, Si Liu* and Xiaomu Wang**

Supporting Information

Perovskite Neuromorphic Engine for Transformer Architectures

Zhenye Zhan[1,5], Yulu Gao[2,5], Yue Liao[3], Weiguang Xie[1], Si Liu[2*], Xiaomu Wang[2,4*]*

[1] Siyuan Laboratory, Guangdong Provincial Engineering Technology Research Center of Vacuum Coating Technologies and New Energy Materials, Department of Physics, Jinan University, Guangzhou, Guangdong, China

[2] Institute of Artificial Intelligence, Beihang University, Beijing, China

[3] Department of Electronic Engineering, The Chinese University of Hong Kong, Hong Kong, China

[4] School of Electronic Science and Engineering, Nanjing University, Nanjing, China

[5] These authors contributed equally to this work

[*]Corresponding authors:
liusi@buaa.edu.cn; wgxie@email.jnu.edu.cn; xiaomu.wang@nju.edu.cn

Table of contents

Note S1 – Device Fabrication and Film Characterization

Figure S1. Optical photos of each step in the preparation of a 4-inch PCU wafer.
Figure S2. Uniformity of the sequential vapor deposited $MAPbI_3$ films.
Figure S3. SEM images of perovskite thin film and devices.

Note S2 – Device Characterization

Figure S4. Mechanism of multistate memristors.
Figure S5. Performance of binary memristors.
Figure S6. The results of 10 cycles I-V characterization performed on 6 devices.

Note S3 – PCU hardware

Table S1. PCU Operation Mode
Figure S7. Circuit diagram.

Note S4 – Comparison of the energy consumption between PCU based hardware and GPU

Table S2. Energy efficiency calculation of PCU based components.
Table S3. Estimation of energy consumption for a transformer block of PCU based hardware and GPU based hardware.
Table S4. Estimation of energy consumption for a transformer block of PCU based memristors with GPU in AD-DA framework.
Figure S8. Energy consumption and energy efficiency.

Note S5 – RGB-T Tracking

Figure S9. RGB-T Tracking with perovskite computing units.

References

Note S1 – Device Fabrication and Film Characterization

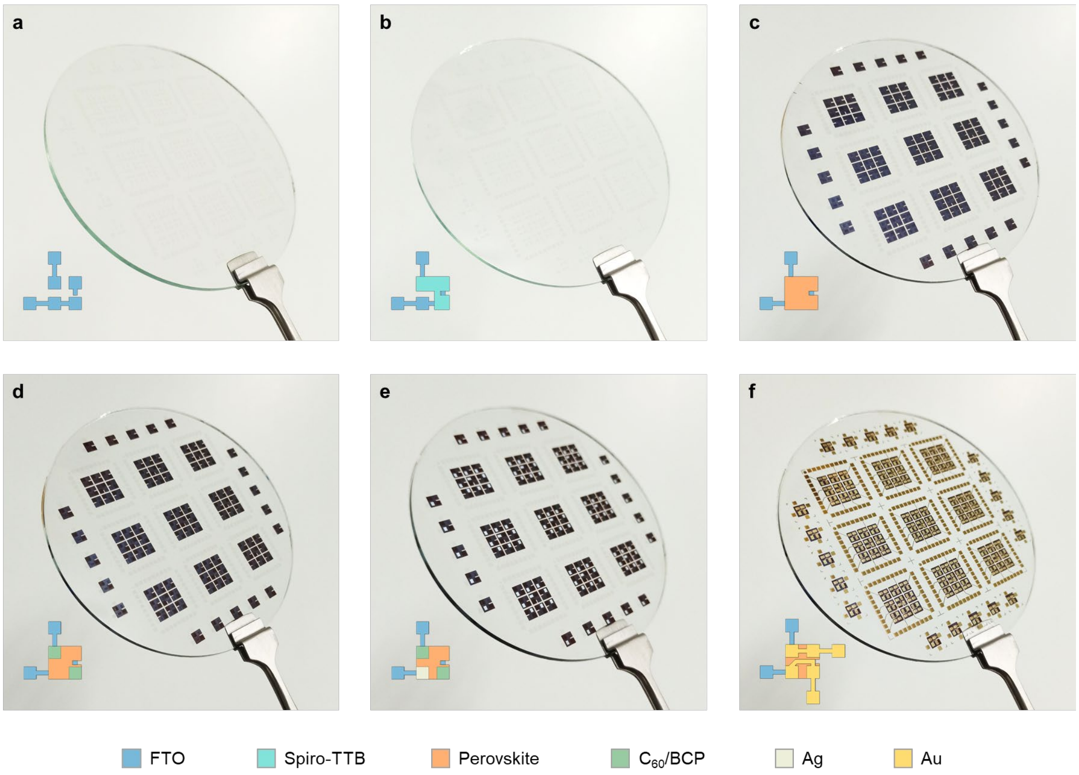

Figure S1. Optical photos of each step in the preparation of a 4-inch PCU wafer. a, The pre-patterned FTO substrate. **b,** The 6 nm-thick layer of Spiro-TTB was firstly deposited by thermal evaporation. **c,** The 250 nm-thick layer of PbI_2 was deposited by thermal evaporation and the perovskite film was prepared using sublimed MAI powder onto the PbI_2 layer and reacted in situ. **d,** The 23 nm of C_{60} and 8 nm of BCP were thermally evaporated onto the perovskite film layer by layer. **e,** The Ag was thermally evaporated. **f,** The Au electrode was finally thermally evaporated.

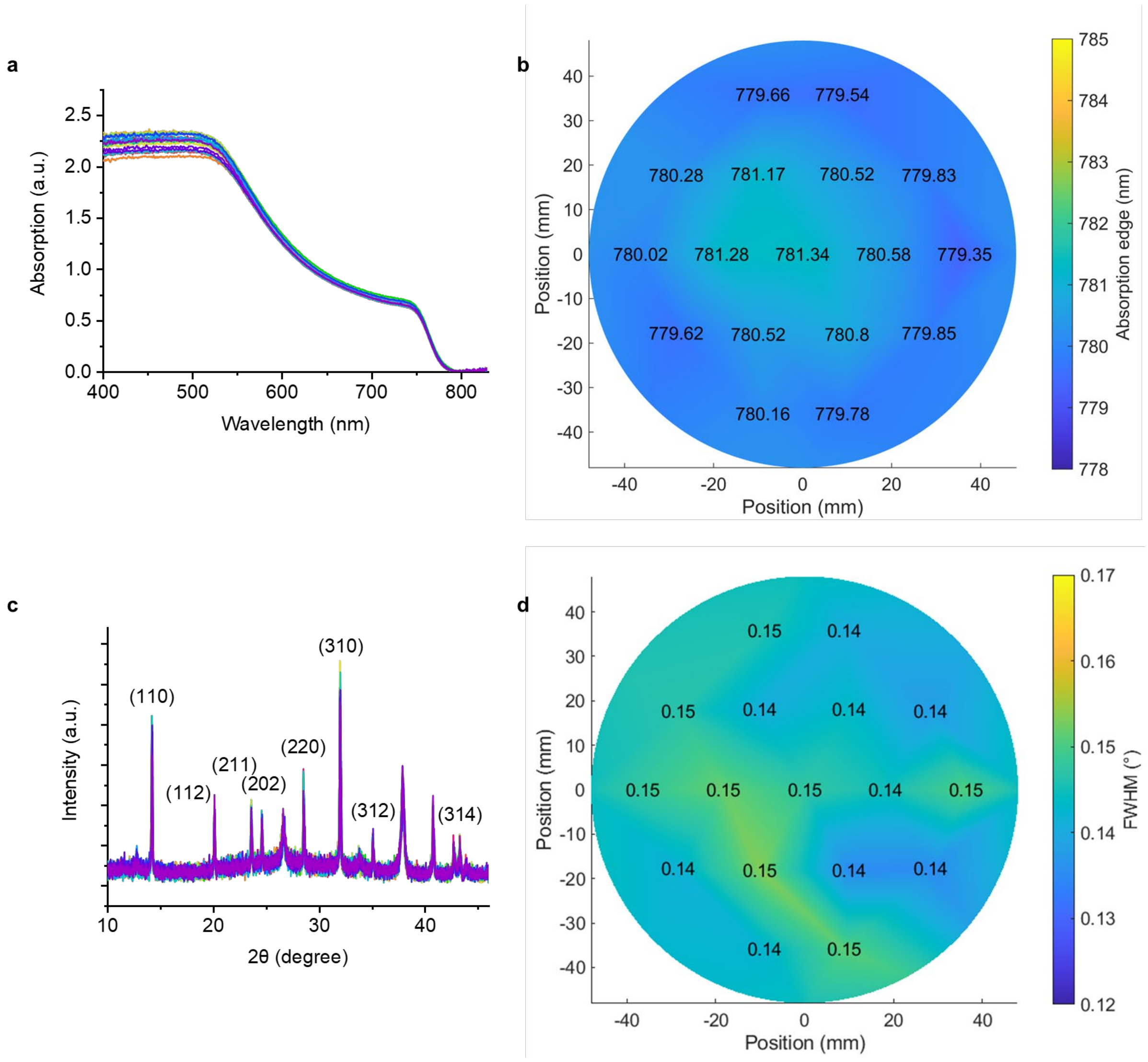

Figure S2. Uniformity of the sequential vapor deposited $MAPbI_3$ films. a, The ultraviolet-visible absorption spectra of $MAPbI_3$ perovskite film at 17 substrate locations on the 4-inch wafer and **b,** the corresponding absorbance edge mapping. The unmeasured positions are filled with linear interpolation. **c,** The XRD result of $MAPbI_3$ perovskite film at 17 substrate locations on the 4-inch wafer. **d,** The mapping of the full width at half maximum of the (310) diffraction peak at 31.9°. The full width at half maximum was fitted with Gaussian function.

The perovskite films prepared by layer-by-layer sequential vapor deposition have high uniformity. Ultraviolet-visible absorption spectra and X-ray diffraction were measured at 17 locations on the 4-inch film. The absorption edge is 780.2±0.6 nm, and the full width at half maximum of the (310) diffraction peak at 31.9° is 0.14±0.01° at 17 positions.

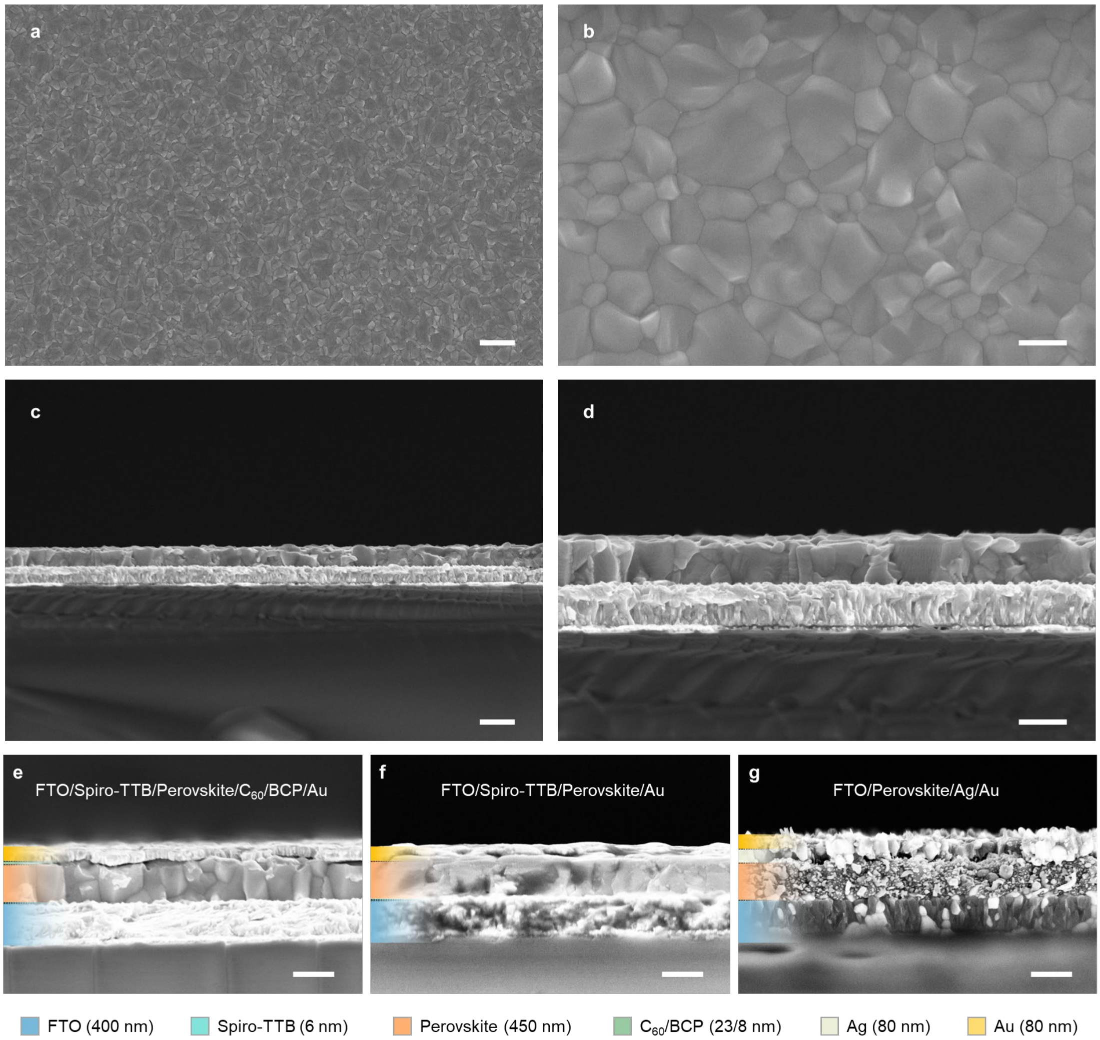

Figure S3. SEM images of perovskite thin film and devices. a,b, Top view images and **c,d,** Cross-sectional images of perovskite thin films with two different magnifications. Scale bar: 1 μm and 400 nm, respectively. Cross-sectional images of **e,** a diode device, **f,** a multi-state memristor, and **g,** a binary memristor. Scale bar: 400 nm.

Here, the diode is fabricated as FTO/Spiro-TTB/Perovskite/C_{60}/BCP/Au structure, in which Spiro-TTB hole-transport and C_{60}/BCP electron-transport layers are inserted between the electrodes and the perovskite. First, these two layers offers suitable energy level alignment to efficiently separate electrons and holes in the perovskite, enable a p-i-n junction with rectification ratio $>10^5$ under ± 1.0V. Second, the layers act as buffer layers during the fabrication. The Spiro-TTB layer facilitates the growth of highly crystalized perovskite. The

C_{60}/BCP layer protects the perovskite surface from the destruction of the deposition of Au. Both of them ensure the fabrication of high quality, smooth, dense and compact perovskite and diode as shown in **Figure S3e**.

The multistate memristor (M1) is constructed as FTO/Spiro-TTB/Perovskite/Au as shown in Figure S3f. Due to the missing of C_{60}/BCP layer, the deposition of Au leads to partially destruction of the perovskite surface, generating iodine vacancy as trap for the tuning of the conductance of the device. Detail mechanism is explained in **Figure S4**.

The binary memristor (M2) is constructed as FTO/Perovskite/Ag. Due to the lack of transporting layer, the perovskite layer is less crystallized in Figure S3g, offering a lot of vacancy channel for the moving of I^-. More importantly, the Ag layer is able to react with the perovskite surface to form a AgI_x surface layer, which serving as a reservoir layer for I^- ions. There ensure the bias tunable connection and disconnection of conductive filament in the perovskite thin film. Detail mechanism is explained in **Figure S5**.

Note S2 – Device Characterization

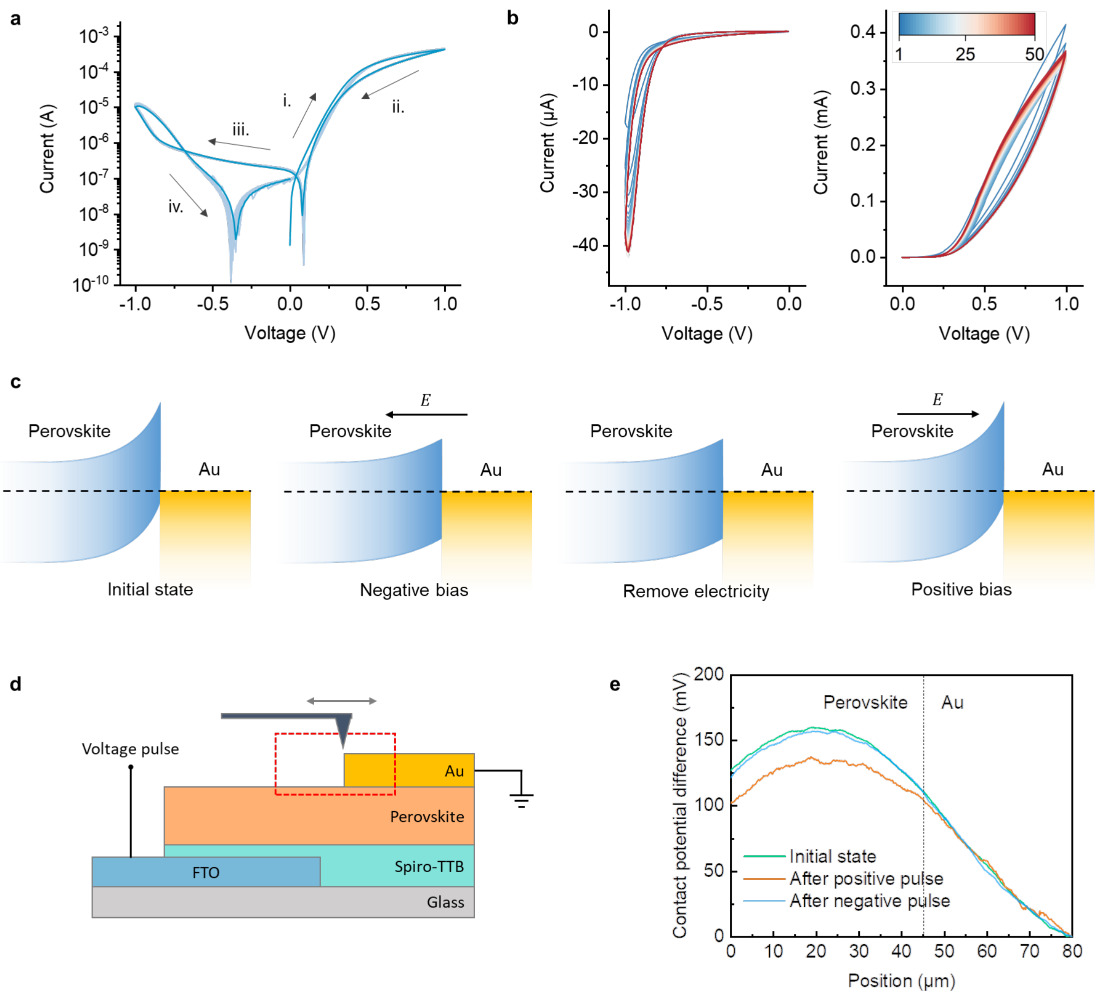

Figure S4. Mechanism of multistate memristors. a, I–V characteristics of 100 voltage sweeping cycles. The arrows indicate the sweeping directions. The I-V curve of the first scan is distinguished by a deep blue color. **b,** Memristive characteristics of the device under the 50 cycles of positive and negative bias scanning. **c,** The energy-band diagrams of multi-states memristors device in different operating states. **d,** Schematic diagram of the device for measuring CPD using Kelvin probe force microscopy. **e,** The CPD results of the perovskite/Au interface under the initial state, after applying a positive pulse, and after applying a negative pulse.

As shown in the energy-band diagrams in **Figure S4c**, there are high concentration of V_I trap states at the interface of $MAPbI_3$/Au. Electrons are trapped at the interface that bend up the

interfacial energy level. When a negative voltage is applied to the FTO side, electrons are detraped. The energy level is bent down, resulting in increased conductivity. After reaching equilibrium, it will form a stable charge doping in the perovskite, thus maintaining a stable conductivity state.[1,2] Conversely, when a positive voltage is applied, electrons will fill the traps, and the barrier at the interface increases, leading to decreased conductivity. Therefore, the interface barrier can be adjusted by the magnitude, polarity, and duration of the applied voltage.

We measured the contact potential difference (CPD) by Kelvin probe force microscope using the device structure in Figure S4d. Figure S4e shows that the measured lateral CPD line profile across the interface of perovskite/Au. The CPD is measured by AFM tip biased condition, it changed inversely to the vacuum level. It's found that the CPD of perovskite decreases when positive pulse is applied. It means that the energy level bent up at positive bias, and vice versa. It is consistent with the energy diagram in Figure S4c.

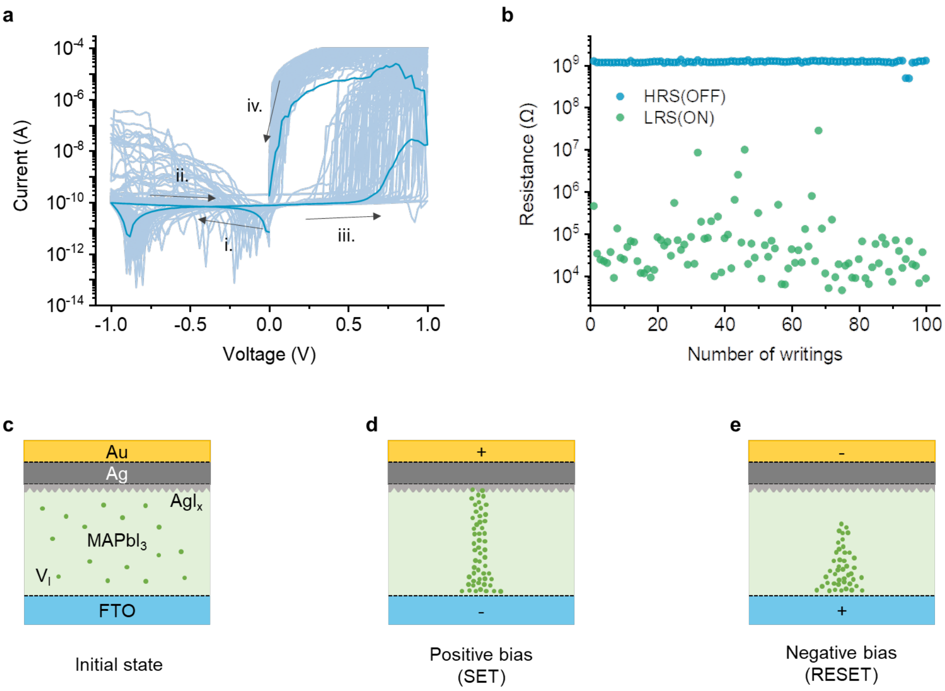

Figure S5. Performance of binary memristors. a, I–V characteristics of 100 voltage sweeping cycles. The arrows indicate the sweeping directions. **b,** Endurance performance with increasing cycles. **c,** The initial state, **d,** SET, and **e,** RESET process.

The consistent resistance switching behavior observed in the FTO/$MAPbI_3$/Ag binary memristor when subjected to electric fields can be attributed to the formation of conductive filaments within the $MAPbI_3$ perovskite material and interface formed between perovskite and silver electrode[3]. When a SET voltage is applied across the binary memristor, iodide ions within the $MAPbI_3$ film drift towards the Ag electrodes, creating iodine vacancies and form the filament as a conductive path (**Fig.S5d**). Due to its high electrochemical activity, Ag can react with I^- ions to form AgI_x, serving as a reservoir layer for I^- ions. This interaction suppresses the diffusion of I^- ions and reduces recombination with iodine vacancies, thereby ensuring the long-term stability of the conductive path[4]. This filament forms between the FTO and Ag electrodes, reducing the overall resistance of the device and switching it to a LRS. When the reversed RESET voltage is applied, the iodide ions drift back and combine with iodine vacancies, breaking the filament and increasing the resistance, switching the device back to a HRS (Fig.S5e). In the experimental measurements, the SET voltage was configured at +1.0V and the RESET voltage at -1.0V to ensure a transition to LRS and HRS, respectively.

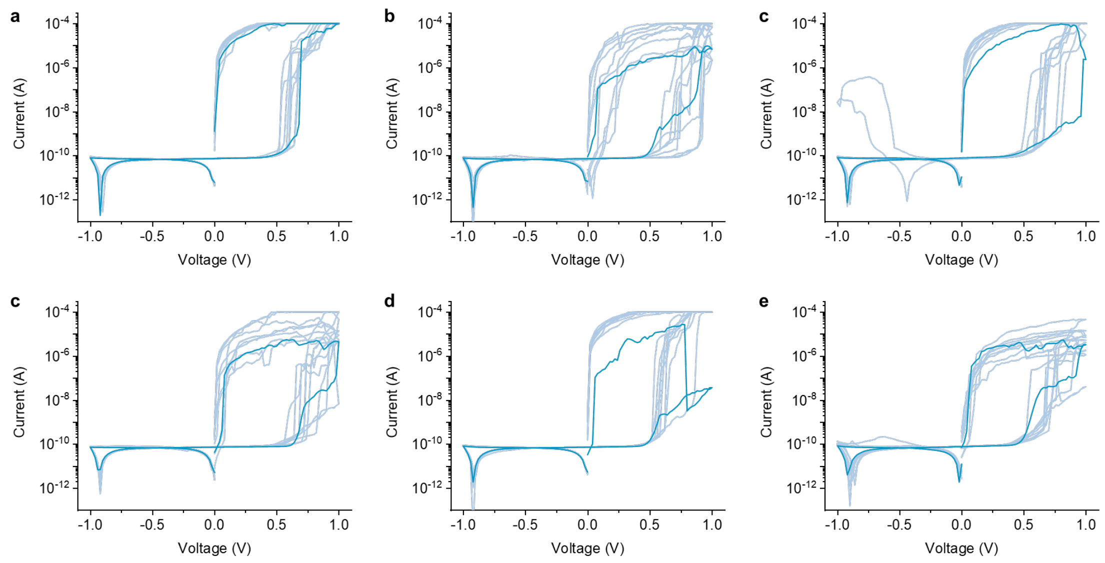

Figure S6. The results of 10 cycles I-V characterization performed on 6 devices.

Note S3 – PCU hardware

Table S1. PCU Operation Mode

Target function	Config voltages	M1 state	M2 state	Results
Linear VMM	Line 1: ISPVA method<br>Line 2: +1.0V SET pulse	G = 1.3 - 84.2 μS tunable	LRS	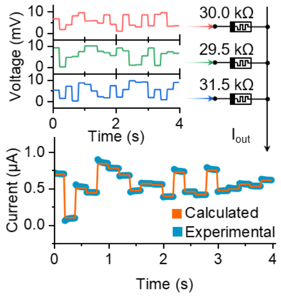
Softplus function	Line 1: ISPVA method<br>Line 2: +1.0V SET pulse	10 kΩ	LRS	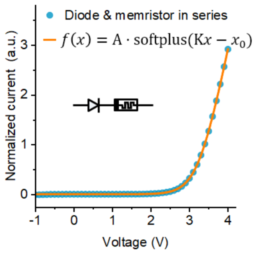
Sigmoid function	Line 1: ISPVA method<br>Line 2: -1.0V RESET pulse	LRS	HRS	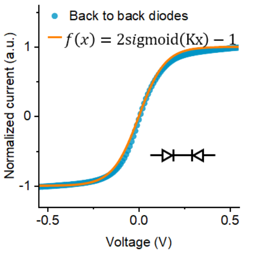

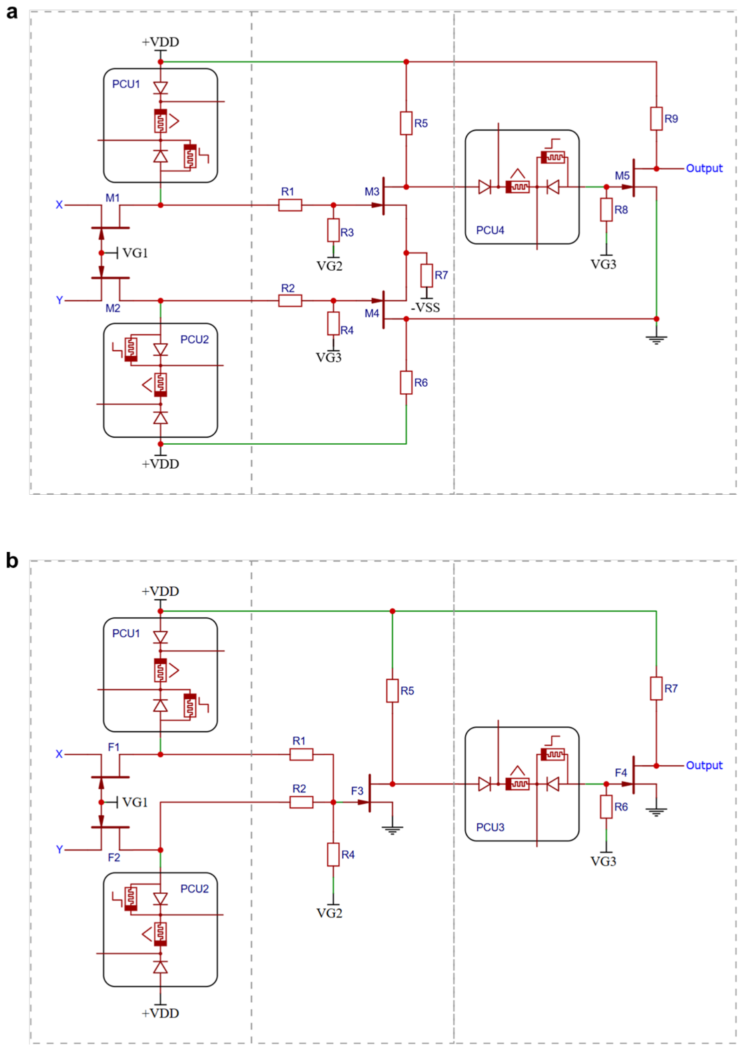

Figure S7. Circuit diagram. Schematic illustrating the interconnection of PCUs to form the **a,** divider and **b,** multiplier. This configuration is accomplished by programming the M1 and M2 to LRS in all the PCUs.

Note S4 – Comparison of the energy consumption between PCU based hardware and GPU

We have computed the energy efficiency for several components, including memristor, exponentiation, multiplication, division, and softplus, as shown in **Table S2**. Based on the resulting energy efficiency of the PCU, we evaluate its energy performance against that of the GPU. The GPU energy efficiency referring to the NVIDIA H100 PCIe, is 0.15 TOPS/W (Tera Operations Per Second per Watt) for general-purpose computing, corresponding to an energy consumption of $E_{GPU} = \frac{805700608 \text{ OPs}}{0.15 \text{ TOPS/W}} = 5.37 \text{ mJ}$. **Table S3** summarizes the number of operations in a transformer block and compares the estimated energy consumption of PCU-based hardware and GPUs. The transformer block under the following configuration: input sequence length = 128, model dimension = 512, and number of attention heads = 8.

In comparison with GPU-based methods, our approach demonstrates a significant advantage in energy efficiency. The power consumption of the GPU (5.37 mJ) is over 50 times higher than that of the PCU based hardware (92.44 μJ) for the same computing task, highlighting the potential for reducing energy costs in large-scale deployments.

Table S4 estimated the power consumption in the above transformer block for the AD-DA framework. Here, we estimate the power consumption of the ADC to be 1 mW and the operating speed to be 10 MHz, resulting in an energy efficiency of $\frac{10 \text{ M OPs}}{0.001 \text{ W}} = 0.01 \text{ TOPS/W}$. The comparison between the energy consumption of a fully PCU based hardware and a hybrid system consisting of a PCU memristor component, ADC, and GPU reveals an advantage for the fully analog approach. The energy consumption of the fully PCU based hardware is 92.44 μJ, whereas the hybrid system consumes 323.52 μJ for the same computation task. By eliminating the need for additional components such as the ADC and GPU, the fully PCU based hardware not only minimizes energy consumption but also simplifies the system architecture, leading to potential cost savings and enhanced performance in energy-sensitive applications. Above comparison of power consumption and energy efficiency is summarized in **Figure S8**.

Table S2. Energy efficiency calculation of PCU based components.

<table>
<tr><th>Component</th><th>Average power
(W)</th><th>Operating
speed
(Hz)</th><th>Number of
operations
(OP)</th><th>Energy efficiency
(TOPS/W)</th></tr>
<tr><td>Memristor</td><td>$3\times3\times8.0\times10^{-9}$ [a]</td><td rowspan="5">50 k [d]</td><td>3×3×2×50 k [b]</td><td>12.50</td></tr>
<tr><td>Exponentiation</td><td>2.7×10^{-8}</td><td>50 k</td><td>1.85</td></tr>
<tr><td>Multiplication</td><td>$3\times2.7\times10^{-8}$ [c]</td><td>2×50 k [b]</td><td>1.24</td></tr>
<tr><td>Division</td><td>$3\times2.7\times10^{-8}$ [c]</td><td>50 k</td><td>0.62</td></tr>
<tr><td>Softplus</td><td>6.6×10^{-7}</td><td>50 k</td><td>0.08</td></tr>
</table>

[a] Consider a 3×3 memristor crossbar network.

[b] Each operation includes multiplication and addition.

[c] Each multiplier and divider require three PCUs.

[d] The frequency at which the system can effectively write data to the corresponding modules and measure the output results, corresponding to a computational latency of 20 μs.

Table S3. Estimation of energy consumption for a transformer block of PCU based hardware and GPU based hardware.

Block in networks		PCU based hardware		GPU (NVIDIA H100)	
Operator	Quantity (OP)	Energy efficiency (TOPS/W)	Energy consumption	Energy efficiency (TOPS/W)	Energy consumption
Memristor	201326592	12.50	16.11 µJ	0.15	5.37 mJ
Multiplication	16777216	1.24	13.59 µJ		
Exponentiation	131072	1.85	70.78 nJ		
Division	1024	0.62	1.66 nJ		
Multiplication	16777216	1.24	13.59 µJ		
Memristor	33554432	12.50	2.68 µJ		
Softplus	262144	0.08	3.45 µJ		
Memristor	536870912	12.50	42.95 µJ		
Total	805700608		92.44 µJ		5.37 mJ

Table S4. Estimation of energy consumption for a transformer block of PCU based memristors with GPU in AD-DA framework.

Block in networks		Hardware	Energy efficiency (TOPS/W)	Energy consumption
Operator	Quantity (OP)			
Memristor	201326592	PCU	12.50	16.11 μJ
	65536	ADC	0.01	6.55 μJ
Multiplication	16777216	GPU	0.15	111.85 μJ
Exponentiation	131072			0.87 μJ
Division	1024			6.83 nJ
Multiplication	16777216			111.85 μJ
	65536	DAC		
Memristor	67108864	PCU	12.50	5.37 μJ
	262144	ADC	0.01	26.21 μJ
Softplus	262144	GPU	0.15	1.75 μJ
Memristor	536870912	PCU	12.50	42.95 μJ
	262144	DAC		
Total	839910400			323.52 μJ

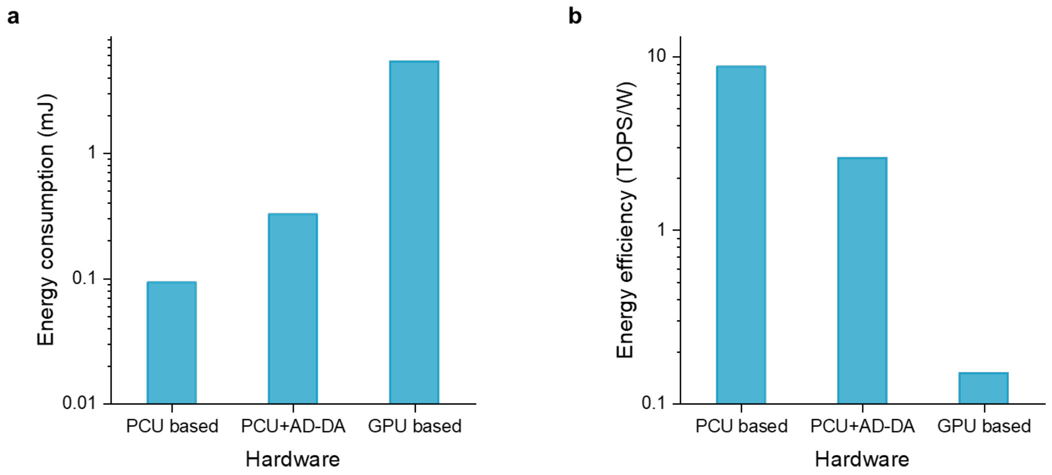

Figure S8. Energy consumption and energy efficiency. a, Energy consumption and **b,** the energy efficiency of the PCU based, PCU based memristors with GPU in AD-DA framework and GPU (NVIDIA H100) based hardware.

Note S5 – RGB-T Tracking

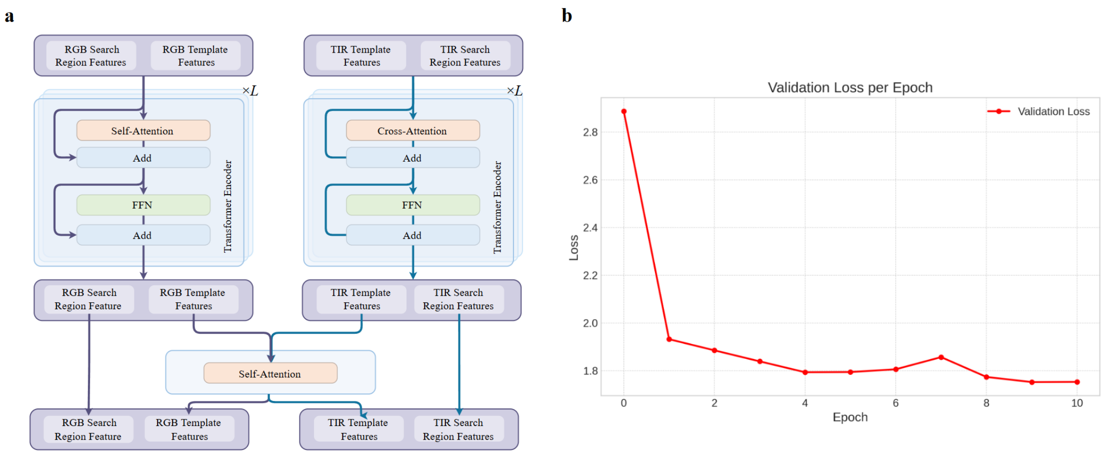

Figure S9. RGB-T Tracking with perovskite computing units. a, The network architecture for the RGB-T Tracking task. **b,** Validation loss per epoch using our Hardware-Aware Offline Fine-Tuning.

Figure S9a illustrates the network architecture for the RGB-T Tracking task, where all operators used in the network can be implemented by our perovskite computing units (PCUs). Figure S9b shows the validation loss per epoch using our Hardware-Aware Offline Fine-Tuning. As seen, the validation loss significantly decreases after fine-tuning. After applying Hardware-Aware Offline Fine-Tuning, the model achieves a final precision of 62.1 and norm precision of 58.7, compared to the original TBSI model, which has a precision of 64.1 and norm precision of 60.0.

References

[1] F. Zhou, Y. Liu, X. Shen, M. Wang, F. Yuan, Y. Chai. Low-Voltage, Optoelectronic $CH_3NH_3PbI_{3-x}Cl_x$ Memory with Integrated Sensing and Logic Operations, *Adv. Funct. Mater.* **2018**, *28*, 1800080. 10.1002/adfm.201800080.

[2] H. Ma, W. Wang, H. Xu, Z. Wang, Y. Tao, P. Chen, W. Liu, X. Zhang, J. Ma, Y. Liu. Interface State-Induced Negative Differential Resistance Observed in Hybrid Perovskite Resistive Switching Memory, *ACS Appl. Mater. Interfaces* **2018**, *10*, 21755. 10.1021/acsami.8b07850.

[3] X. Song, J. Zhang, Y. Qian, Z. Xia, J. Chen, H. Yin, J. Liu, L. Feng, T. Liu, Z. Zhu, Y. Hua, Y. Liu, J. Yuan, F. Ge, D. Zhou, M. Li, Y. Hang, F. Wang, T. Qin, L. Wang. Simultaneous resistance switching and rectifying effects in a single hybrid perovskite, *InfoMat* **2024**, *6*, e12562. 10.1002/inf2.12562.

[4] X. Zhu, J. Lee, W. D. Lu. Iodine Vacancy Redistribution in Organic-Inorganic Halide Perovskite Films and Resistive Switching Effects, *Adv. Mater.* **2017**, *29*. 10.1002/adma.201700527

.
